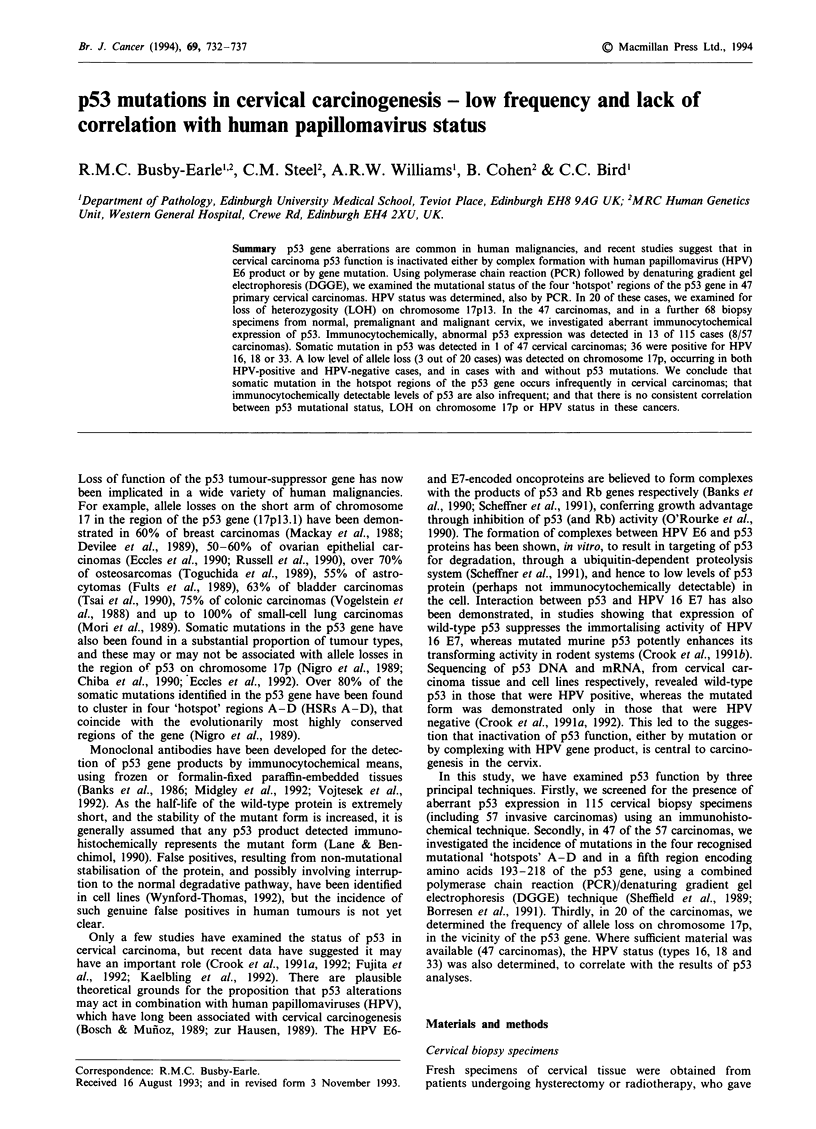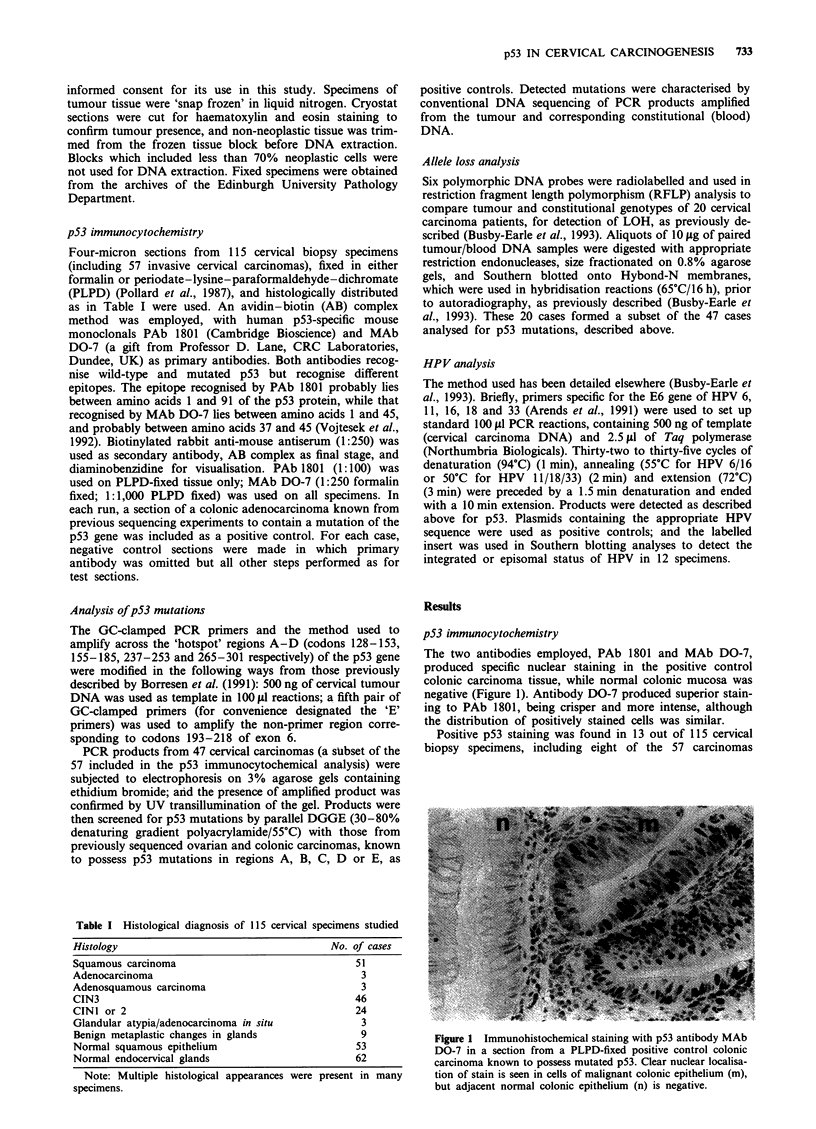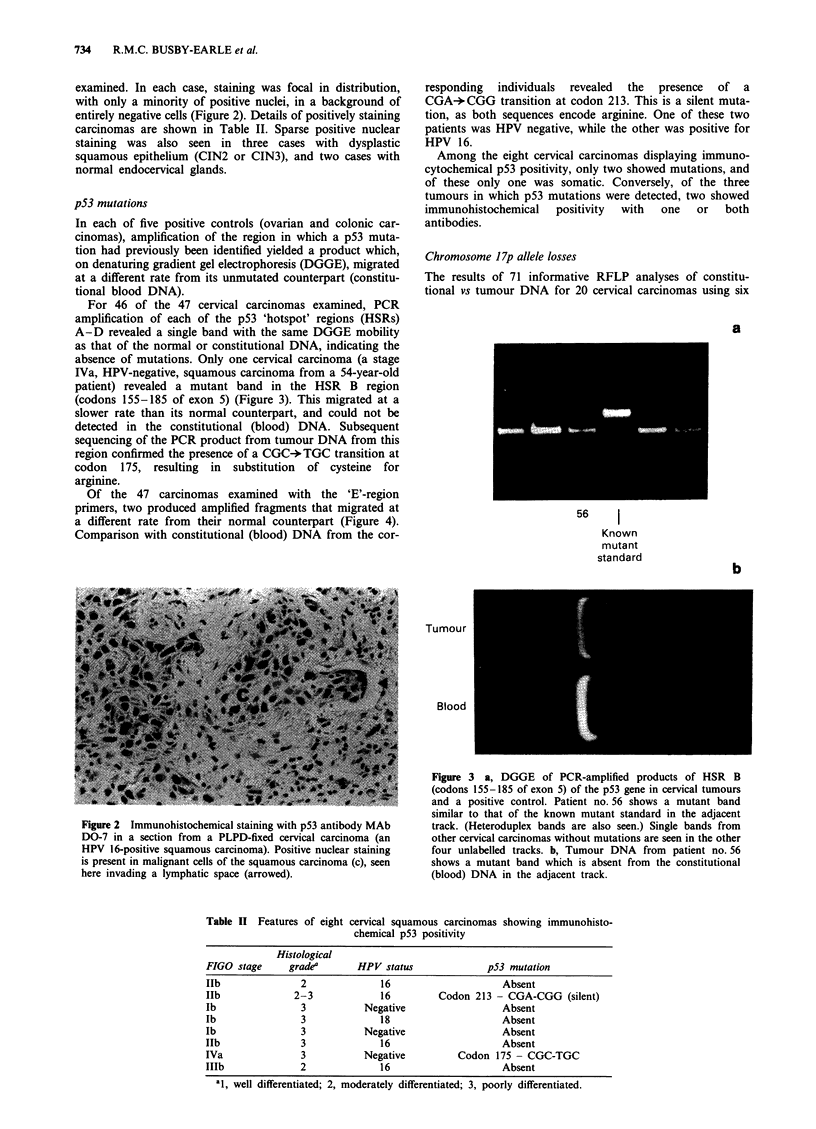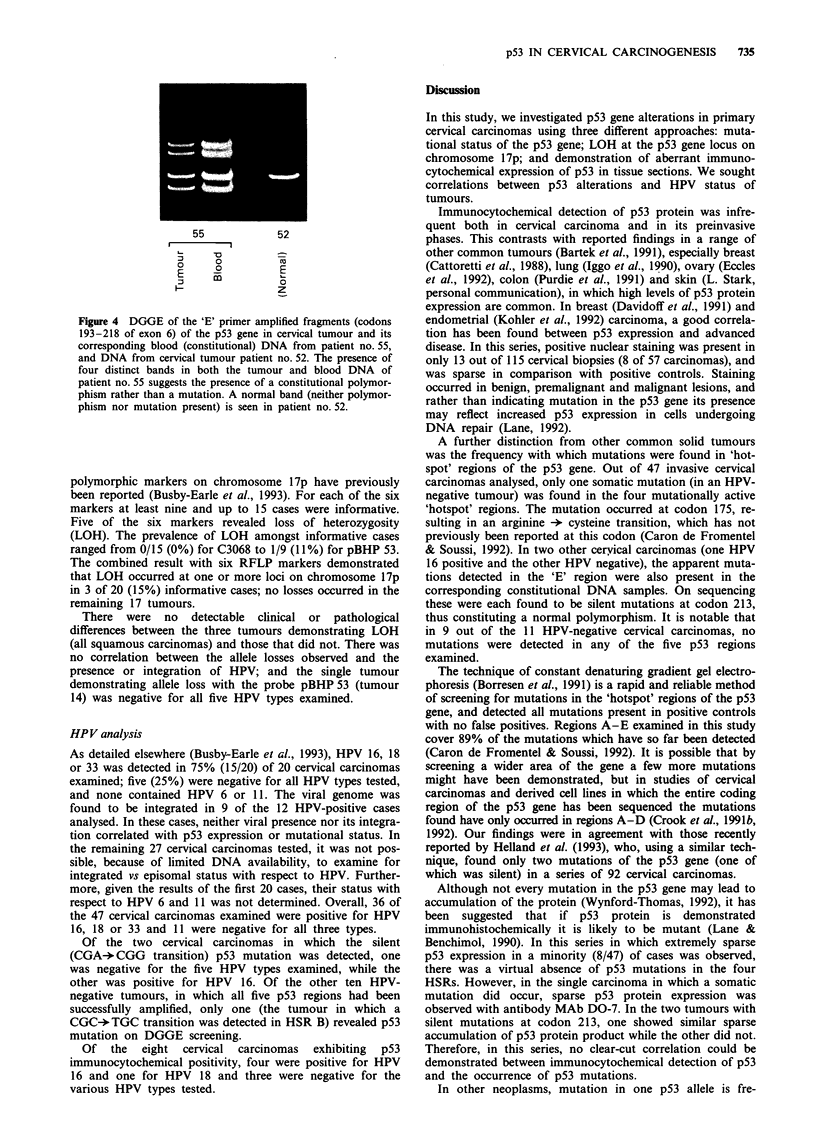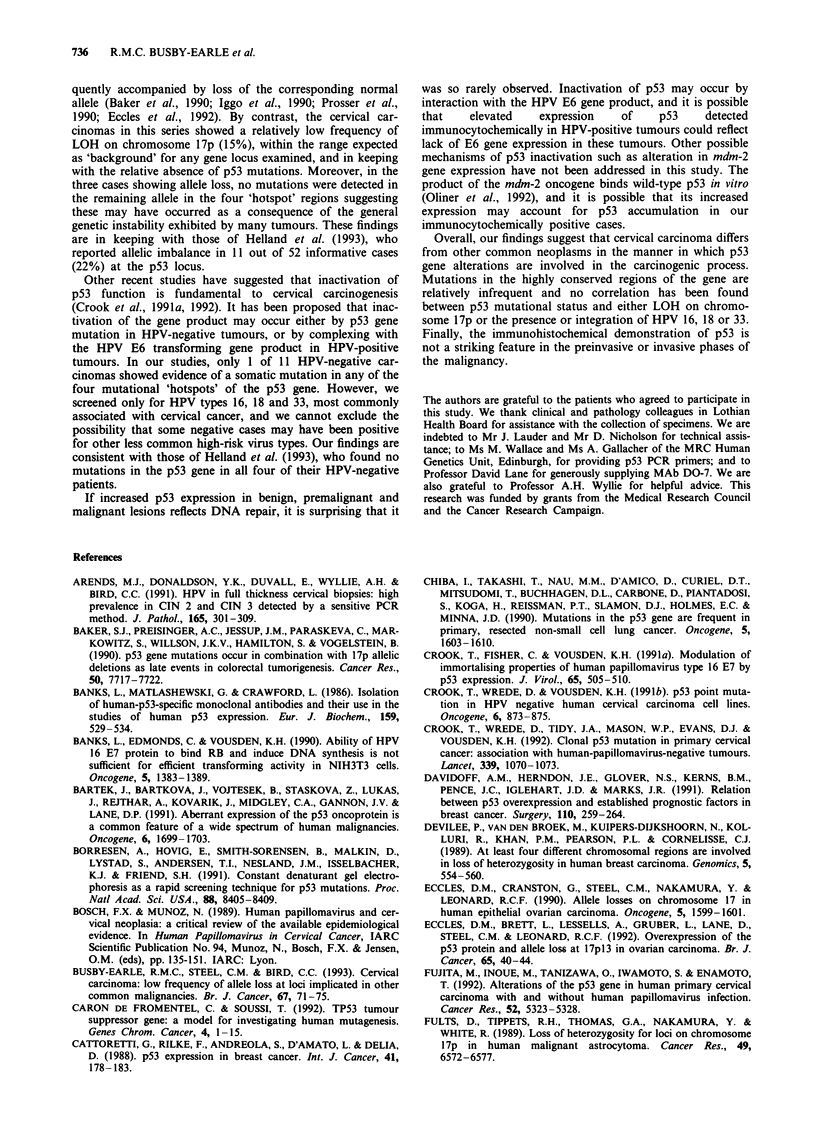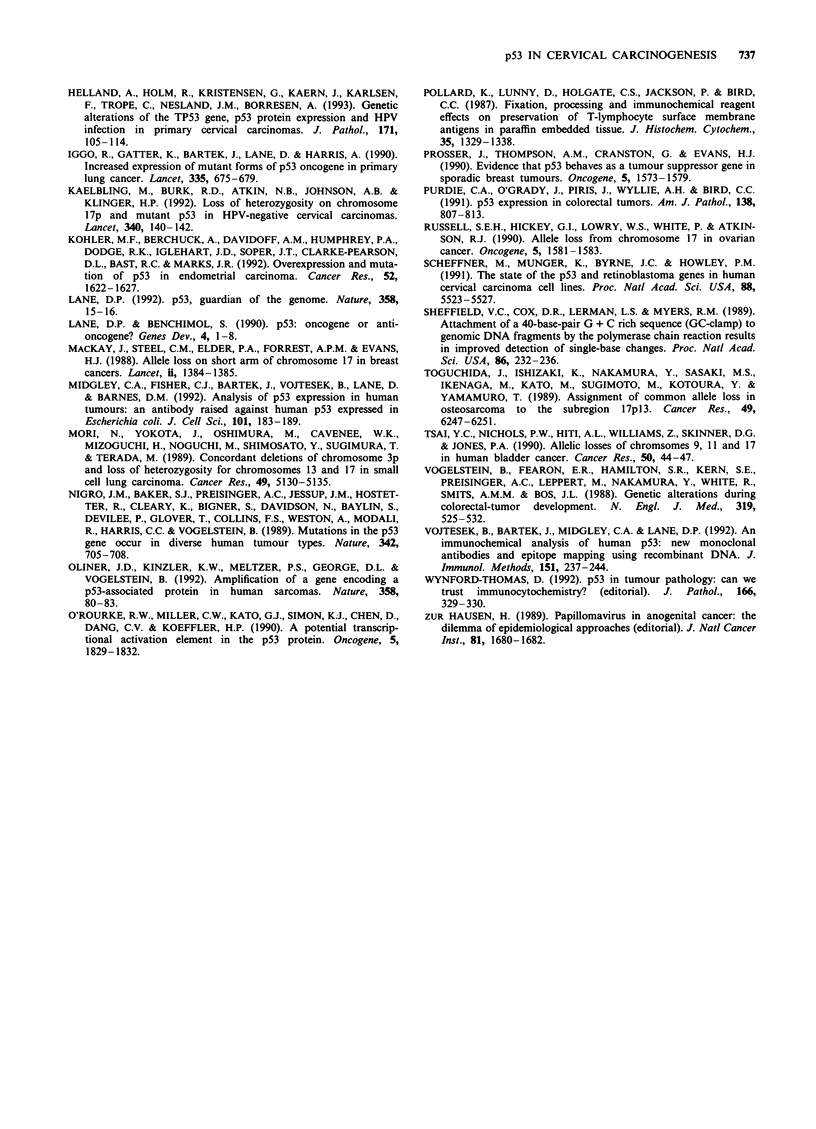# p53 mutations in cervical carcinogenesis--low frequency and lack of correlation with human papillomavirus status.

**DOI:** 10.1038/bjc.1994.138

**Published:** 1994-04

**Authors:** R. M. Busby-Earle, C. M. Steel, A. R. Williams, B. Cohen, C. C. Bird

**Affiliations:** Department of Pathology, Edinburgh University Medical School, UK.

## Abstract

**Images:**


 © Macmillan Press Ltd., 1994

### p53 mutations in cervical carcinogenesis – low frequency and lack of correlation with human papillomavirus status

R.M.C. Busby-Earle[1,2], C.M. Steel[2], A.R.W. Williams[1], B. Cohen[2] & C.C. Bird[1]

[1]*Department of Pathology, Edinburgh University Medical School, Teviot Place, Edinburgh EH8 9AG UK;* [2]*MRC Human Genetics Unit, Western General Hospital, Crewe Rd, Edinburgh EH4 2XU, UK.*

**Summary** p53 gene aberrations are common in human malignancies, and recent studies suggest that in cervical carcinoma p53 function is inactivated either by complex formation with human papillomavirus (HPV) E6 product or by gene mutation. Using polymerase chain reaction (PCR) followed by denaturing gradient gel electrophoresis (DGGE), we examined the mutational status of the four 'hotspot' regions of the p53 gene in 47 primary cervical carcinomas. HPV status was determined, also by PCR. In 20 of these cases, we examined for loss of heterozygosity (LOH) on chromosome 17p13. In the 47 carcinomas, and in a further 68 biopsy specimens from normal, premalignant and malignant cervix, we investigated aberrant immunocytochemical expression of p53. Immunocytochemically, abnormal p53 expression was detected in 13 of 115 cases (8/57 carcinomas). Somatic mutation in p53 was detected in 1 of 47 cervical carcinomas; 36 were positive for HPV 16, 18 or 33. A low level of allele loss (3 out of 20 cases) was detected on chromosome 17p, occurring in both HPV-positive and HPV-negative cases, and in cases with and without p53 mutations. We conclude that somatic mutation in the hotspot regions of the p53 gene occurs infrequently in cervical carcinomas; that immunocytochemically detectable levels of p53 are also infrequent; and that there is no consistent correlation between p53 mutational status, LOH on chromosome 17p or HPV status in these cancers.

Loss of function of the p53 tumour-suppressor gene has now been implicated in a wide variety of human malignancies. For example, allele losses on the short arm of chromosome 17 in the region of the p53 gene (17p13.1) have been demonstrated in 60% of breast carcinomas (Mackay *et al.*, 1988; Devilee *et al.*, 1989), 50–60% of ovarian epithelial carcinomas (Eccles *et al.*, 1990; Russell *et al.*, 1990), over 70% of osteosarcomas (Toguchida *et al.*, 1989), 55% of astrocytomas (Fults *et al.*, 1989), 63% of bladder carcinomas (Tsai *et al.*, 1990), 75% of colonic carcinomas (Vogelstein *et al.*, 1988) and up to 100% of small-cell lung carcinomas (Mori *et al.*, 1989). Somatic mutations in the p53 gene have also been found in a substantial proportion of tumour types, and these may or may not be associated with allele losses in the region of p53 on chromosome 17p (Nigro *et al.*, 1989; Chiba *et al.*, 1990; Eccles *et al.*, 1992). Over 80% of the somatic mutations identified in the p53 gene have been found to cluster in four 'hotspot' regions A–D (HSRs A–D), that coincide with the evolutionarily most highly conserved regions of the gene (Nigro *et al.*, 1989).

Monoclonal antibodies have been developed for the detection of p53 gene products by immunocytochemical means, using frozen or formalin-fixed paraffin-embedded tissues (Banks *et al.*, 1986; Midgley *et al.*, 1992; Vojtesek *et al.*, 1992). As the half-life of the wild-type protein is extremely short, and the stability of the mutant form is increased, it is generally assumed that any p53 product detected immunohistochemically represents the mutant form (Lane & Benchimol, 1990). False positives, resulting from non-mutational stabilisation of the protein, and possibly involving interruption to the normal degradative pathway, have been identified in cell lines (Wynford-Thomas, 1992), but the incidence of such genuine false positives in human tumours is not yet clear.

Only a few studies have examined the status of p53 in cervical carcinoma, but recent data have suggested it may have an important role (Crook *et al.*, 1991*a*, 1992; Fujita *et al.*, 1992; Kaelbling *et al.*, 1992). There are plausible theoretical grounds for the proposition that p53 alterations may act in combination with human papillomaviruses (HPV), which have long been associated with cervical carcinogenesis (Bosch & Muñoz, 1989; zur Hausen, 1989). The HPV E6- and E7-encoded oncoproteins are believed to form complexes with the products of p53 and Rb genes respectively (Banks *et al.*, 1990; Scheffner *et al.*, 1991), conferring growth advantage through inhibition of p53 (and Rb) activity (O'Rourke *et al.*, 1990). The formation of complexes between HPV E6 and p53 proteins has been shown, *in vitro*, to result in targeting of p53 for degradation, through a ubiquitin-dependent proteolysis system (Scheffner *et al.*, 1991), and hence to low levels of p53 protein (perhaps not immunocytochemically detectable) in the cell. Interaction between p53 and HPV 16 E7 has also been demonstrated, in studies showing that expression of wild-type p53 suppresses the immortalising activity of HPV 16 E7, whereas mutated murine p53 potently enhances its transforming activity in rodent systems (Crook *et al.*, 1991*b*). Sequencing of p53 DNA and mRNA, from cervical carcinoma tissue and cell lines respectively, revealed wild-type p53 in those that were HPV positive, whereas the mutated form was demonstrated only in those that were HPV negative (Crook *et al.*, 1991*a*, 1992). This led to the suggestion that inactivation of p53 function, either by mutation or by complexing with HPV gene product, is central to carcinogenesis in the cervix.

In this study, we have examined p53 function by three principal techniques. Firstly, we screened for the presence of aberrant p53 expression in 115 cervical biopsy specimens (including 57 invasive carcinomas) using an immunohistochemical technique. Secondly, in 47 of the 57 carcinomas, we investigated the incidence of mutations in the four recognised mutational 'hotspots' A–D and in a fifth region encoding amino acids 193–218 of the p53 gene, using a combined polymerase chain reaction (PCR)/denaturing gradient gel electrophoresis (DGGE) technique (Sheffield *et al.*, 1989; Borresen *et al.*, 1991). Thirdly, in 20 of the carcinomas, we determined the frequency of allele loss on chromosome 17p, in the vicinity of the p53 gene. Where sufficient material was available (47 carcinomas), the HPV status (types 16, 18 and 33) was also determined, to correlate with the results of p53 analyses.

### Materials and methods

### *Cervical biopsy specimens*

Fresh specimens of cervical tissue were obtained from patients undergoing hysterectomy or radiotherapy, who gave

Correspondence: R.M.C. Busby-Earle.
Received 16 August 1993; and in revised form 3 November 1993.

informed consent for its use in this study. Specimens of tumour tissue were 'snap frozen' in liquid nitrogen. Cryostat sections were cut for haematoxylin and eosin staining to confirm tumour presence, and non-neoplastic tissue was trimmed from the frozen tissue block before DNA extraction. Blocks which included less than 70% neoplastic cells were not used for DNA extraction. Fixed specimens were obtained from the archives of the Edinburgh University Pathology Department.

*p53 immunocytochemistry*

Four-micron sections from 115 cervical biopsy specimens (including 57 invasive cervical carcinomas), fixed in either formalin or periodate–lysine–paraformaldehyde–dichromate (PLPD) (Pollard *et al.*, 1987), and histologically distributed as in Table I were used. An avidin–biotin (AB) complex method was employed, with human p53-specific mouse monoclonals PAb 1801 (Cambridge Bioscience) and MAb DO-7 (a gift from Professor D. Lane, CRC Laboratories, Dundee, UK) as primary antibodies. Both antibodies recognise wild-type and mutated p53 but recognise different epitopes. The epitope recognised by PAb 1801 probably lies between amino acids 1 and 91 of the p53 protein, while that recognised by MAb DO-7 lies between amino acids 1 and 45, and probably between amino acids 37 and 45 (Vojtesek *et al.*, 1992). Biotinylated rabbit anti-mouse antiserum (1:250) was used as secondary antibody, AB complex as final stage, and diaminobenzidine for visualisation. PAb 1801 (1:100) was used on PLPD-fixed tissue only; MAb DO-7 (1:250 formalin fixed; 1:1,000 PLPD fixed) was used on all specimens. In each run, a section of a colonic adenocarcinoma known from previous sequencing experiments to contain a mutation of the p53 gene was included as a positive control. For each case, negative control sections were made in which primary antibody was omitted but all other steps performed as for test sections.

*Analysis of p53 mutations*

The GC-clamped PCR primers and the method used to amplify across the 'hotspot' regions A–D (codons 128–153, 155–185, 237–253 and 265–301 respectively) of the p53 gene were modified in the following ways from those previously described by Borresen *et al.* (1991): 500 ng of cervical tumour DNA was used as template in 100 µl reactions; a fifth pair of GC-clamped primers (for convenience designated the 'E' primers) was used to amplify the non-primer region corresponding to codons 193–218 of exon 6.

PCR products from 47 cervical carcinomas (a subset of the 57 included in the p53 immunocytochemical analysis) were subjected to electrophoresis on 3% agarose gels containing ethidium bromide; and the presence of amplified product was confirmed by UV transillumination of the gel. Products were then screened for p53 mutations by parallel DGGE (30–80% denaturing gradient polyacrylamide/55°C) with those from previously sequenced ovarian and colonic carcinomas, known to possess p53 mutations in regions A, B, C, D or E, as positive controls. Detected mutations were characterised by conventional DNA sequencing of PCR products amplified from the tumour and corresponding constitutional (blood) DNA.

**Table I** Histological diagnosis of 115 cervical specimens studied

| *Histology* | *No. of cases* |
|---|---|
| Squamous carcinoma | 51 |
| Adenocarcinoma | 3 |
| Adenosquamous carcinoma | 3 |
| CIN3 | 46 |
| CIN1 or 2 | 24 |
| Glandular atypia/adenocarcinoma *in situ* | 3 |
| Benign metaplastic changes in glands | 9 |
| Normal squamous epithelium | 53 |
| Normal endocervical glands | 62 |

Note: Multiple histological appearances were present in many specimens.

*Allele loss analysis*

Six polymorphic DNA probes were radiolabelled and used in restriction fragment length polymorphism (RFLP) analysis to compare tumour and constitutional genotypes of 20 cervical carcinoma patients, for detection of LOH, as previously described (Busby-Earle *et al.*, 1993). Aliquots of 10 µg of paired tumour/blood DNA samples were digested with appropriate restriction endonucleases, size fractionated on 0.8% agarose gels, and Southern blotted onto Hybond-N membranes, which were used in hybridisation reactions (65°C/16 h), prior to autoradiography, as previously described (Busby-Earle *et al.*, 1993). These 20 cases formed a subset of the 47 cases analysed for p53 mutations, described above.

*HPV analysis*

The method used has been detailed elsewhere (Busby-Earle *et al.*, 1993). Briefly, primers specific for the E6 gene of HPV 6, 11, 16, 18 and 33 (Arends *et al.*, 1991) were used to set up standard 100 µl PCR reactions, containing 500 ng of template (cervical carcinoma DNA) and 2.5 µl of *Taq* polymerase (Northumbria Biologicals). Thirty-two to thirty-five cycles of denaturation (94°C) (1 min), annealing (55°C for HPV 6/16 or 50°C for HPV 11/18/33) (2 min) and extension (72°C) (3 min) were preceded by a 1.5 min denaturation and ended with a 10 min extension. Products were detected as described above for p53. Plasmids containing the appropriate HPV sequence were used as positive controls; and the labelled insert was used in Southern blotting analyses to detect the integrated or episomal status of HPV in 12 specimens.

### Results

*p53 immunocytochemistry*

The two antibodies employed, PAb 1801 and MAb DO-7, produced specific nuclear staining in the positive control colonic carcinoma tissue, while normal colonic mucosa was negative (Figure 1). Antibody DO-7 produced superior staining to PAb 1801, being crisper and more intense, although the distribution of positively stained cells was similar.

Positive p53 staining was found in 13 out of 115 cervical biopsy specimens, including eight of the 57 carcinomas

**Figure 1** Immunohistochemical staining with p53 antibody MAb DO-7 in a section from a PLPD-fixed positive control colonic carcinoma known to possess mutated p53. Clear nuclear localisation of stain is seen in cells of malignant colonic epithelium (m), but adjacent normal colonic epithelium (n) is negative.

examined. In each case, staining was focal in distribution, with only a minority of positive nuclei, in a background of entirely negative cells (Figure 2). Details of positively staining carcinomas are shown in Table II. Sparse positive nuclear staining was also seen in three cases with dysplastic squamous epithelium (CIN2 or CIN3), and two cases with normal endocervical glands.

*p53 mutations*

In each of five positive controls (ovarian and colonic carcinomas), amplification of the region in which a p53 mutation had previously been identified yielded a product which, on denaturing gradient gel electrophoresis (DGGE), migrated at a different rate from its unmutated counterpart (constitutional blood DNA).

For 46 of the 47 cervical carcinomas examined, PCR amplification of each of the p53 'hotspot' regions (HSRs) A–D revealed a single band with the same DGGE mobility as that of the normal or constitutional DNA, indicating the absence of mutations. Only one cervical carcinoma (a stage IVa, HPV-negative, squamous carcinoma from a 54-year-old patient) revealed a mutant band in the HSR B region (codons 155–185 of exon 5) (Figure 3). This migrated at a slower rate than its normal counterpart, and could not be detected in the constitutional (blood) DNA. Subsequent sequencing of the PCR product from tumour DNA from this region confirmed the presence of a CGC→TGC transition at codon 175, resulting in substitution of cysteine for arginine.

Of the 47 carcinomas examined with the 'E'-region primers, two produced amplified fragments that migrated at a different rate from their normal counterpart (Figure 4). Comparison with constitutional (blood) DNA from the corresponding individuals revealed the presence of a CGA→CGG transition at codon 213. This is a silent mutation, as both sequences encode arginine. One of these two patients was HPV negative, while the other was positive for HPV 16.

Among the eight cervical carcinomas displaying immunocytochemical p53 positivity, only two showed mutations, and of these only one was somatic. Conversely, of the three tumours in which p53 mutations were detected, two showed immunohistochemical positivity with one or both antibodies.

*Chromosome 17p allele losses*

The results of 71 informative RFLP analyses of constitutional *vs* tumour DNA for 20 cervical carcinomas using six

**Figure 2** Immunohistochemical staining with p53 antibody MAb DO-7 in a section from a PLPD-fixed cervical carcinoma (an HPV 16-positive squamous carcinoma). Positive nuclear staining is present in malignant cells of the squamous carcinoma (c), seen here invading a lymphatic space (arrowed).

**Figure 3** **a**, DGGE of PCR-amplified products of HSR B (codons 155–185 of exon 5) of the p53 gene in cervical tumours and a positive control. Patient no. 56 shows a mutant band similar to that of the known mutant standard in the adjacent track. (Heteroduplex bands are also seen.) Single bands from other cervical carcinomas without mutations are seen in the other four unlabelled tracks. **b**, Tumour DNA from patient no. 56 shows a mutant band which is absent from the constitutional (blood) DNA in the adjacent track.

**Table II** Features of eight cervical squamous carcinomas showing immunohistochemical p53 positivity

| *FIGO stage* | *Histological grade*[a] | *HPV status* | *p53 mutation* |
|---|---|---|---|
| IIb | 2 | 16 | Absent |
| IIb | 2–3 | 16 | Codon 213 – CGA-CGG (silent) |
| Ib | 3 | Negative | Absent |
| Ib | 3 | 18 | Absent |
| Ib | 3 | Negative | Absent |
| IIb | 3 | 16 | Absent |
| IVa | 3 | Negative | Codon 175 – CGC-TGC |
| IIIb | 2 | 16 | Absent |

[a]1, well differentiated; 2, moderately differentiated; 3, poorly differentiated.

**Figure 4** DGGE of the 'E' primer amplified fragments (codons 193–218 of exon 6) of the p53 gene in cervical tumour and its corresponding blood (constitutional) DNA from patient no. 55, and DNA from cervical tumour patient no. 52. The presence of four distinct bands in both the tumour and blood DNA of patient no. 55 suggests the presence of a constitutional polymorphism rather than a mutation. A normal band (neither polymorphism nor mutation present) is seen in patient no. 52.

polymorphic markers on chromosome 17p have previously been reported (Busby-Earle *et al.*, 1993). For each of the six markers at least nine and up to 15 cases were informative. Five of the six markers revealed loss of heterozygosity (LOH). The prevalence of LOH amongst informative cases ranged from 0/15 (0%) for C3068 to 1/9 (11%) for pBHP 53. The combined result with six RFLP markers demonstrated that LOH occurred at one or more loci on chromosome 17p in 3 of 20 (15%) informative cases; no losses occurred in the remaining 17 tumours.

There were no detectable clinical or pathological differences between the three tumours demonstrating LOH (all squamous carcinomas) and those that did not. There was no correlation between the allele losses observed and the presence or integration of HPV; and the single tumour demonstrating allele loss with the probe pBHP 53 (tumour 14) was negative for all five HPV types examined.

### *HPV analysis*

As detailed elsewhere (Busby-Earle *et al.*, 1993), HPV 16, 18 or 33 was detected in 75% (15/20) of 20 cervical carcinomas examined; five (25%) were negative for all HPV types tested, and none contained HPV 6 or 11. The viral genome was found to be integrated in 9 of the 12 HPV-positive cases analysed. In these cases, neither viral presence nor its integration correlated with p53 expression or mutational status. In the remaining 27 cervical carcinomas tested, it was not possible, because of limited DNA availability, to examine for integrated *vs* episomal status with respect to HPV. Furthermore, given the results of the first 20 cases, their status with respect to HPV 6 and 11 was not determined. Overall, 36 of the 47 cervical carcinomas examined were positive for HPV 16, 18 or 33 and 11 were negative for all three types.

Of the two cervical carcinomas in which the silent (CGA→CGG transition) p53 mutation was detected, one was negative for the five HPV types examined, while the other was positive for HPV 16. Of the other ten HPV-negative tumours, in which all five p53 regions had been successfully amplified, only one (the tumour in which a CGC→TGC transition was detected in HSR B) revealed p53 mutation on DGGE screening.

Of the eight cervical carcinomas exhibiting p53 immunocytochemical positivity, four were positive for HPV 16 and one for HPV 18 and three were negative for the various HPV types tested.

### Discussion

In this study, we investigated p53 gene alterations in primary cervical carcinomas using three different approaches: mutational status of the p53 gene; LOH at the p53 gene locus on chromosome 17p; and demonstration of aberrant immunocytochemical expression of p53 in tissue sections. We sought correlations between p53 alterations and HPV status of tumours.

Immunocytochemical detection of p53 protein was infrequent both in cervical carcinoma and in its preinvasive phases. This contrasts with reported findings in a range of other common tumours (Bartek *et al.*, 1991), especially breast (Cattoretti *et al.*, 1988), lung (Iggo *et al.*, 1990), ovary (Eccles *et al.*, 1992), colon (Purdie *et al.*, 1991) and skin (L. Stark, personal communication), in which high levels of p53 protein expression are common. In breast (Davidoff *et al.*, 1991) and endometrial (Kohler *et al.*, 1992) carcinoma, a good correlation has been found between p53 expression and advanced disease. In this series, positive nuclear staining was present in only 13 out of 115 cervical biopsies (8 of 57 carcinomas), and was sparse in comparison with positive controls. Staining occurred in benign, premalignant and malignant lesions, and rather than indicating mutation in the p53 gene its presence may reflect increased p53 expression in cells undergoing DNA repair (Lane, 1992).

A further distinction from other common solid tumours was the frequency with which mutations were found in 'hotspot' regions of the p53 gene. Out of 47 invasive cervical carcinomas analysed, only one somatic mutation (in an HPV-negative tumour) was found in the four mutationally active 'hotspot' regions. The mutation occurred at codon 175, resulting in an arginine → cysteine transition, which has not previously been reported at this codon (Caron de Fromentel & Soussi, 1992). In two other cervical carcinomas (one HPV 16 positive and the other HPV negative), the apparent mutations detected in the 'E' region were also present in the corresponding constitutional DNA samples. On sequencing these were each found to be silent mutations at codon 213, thus constituting a normal polymorphism. It is notable that in 9 out of the 11 HPV-negative cervical carcinomas, no mutations were detected in any of the five p53 regions examined.

The technique of constant denaturing gradient gel electrophoresis (Borresen *et al.*, 1991) is a rapid and reliable method of screening for mutations in the 'hotspot' regions of the p53 gene, and detected all mutations present in positive controls with no false positives. Regions A–E examined in this study cover 89% of the mutations which have so far been detected (Caron de Fromentel & Soussi, 1992). It is possible that by screening a wider area of the gene a few more mutations might have been demonstrated, but in studies of cervical carcinomas and derived cell lines in which the entire coding region of the p53 gene has been sequenced the mutations found have only occurred in regions A–D (Crook *et al.*, 1991*b*, 1992). Our findings were in agreement with those recently reported by Helland *et al.* (1993), who, using a similar technique, found only two mutations of the p53 gene (one of which was silent) in a series of 92 cervical carcinomas.

Although not every mutation in the p53 gene may lead to accumulation of the protein (Wynford-Thomas, 1992), it has been suggested that if p53 protein is demonstrated immunohistochemically it is likely to be mutant (Lane & Benchimol, 1990). In this series in which extremely sparse p53 expression in a minority (8/47) of cases was observed, there was a virtual absence of p53 mutations in the four HSRs. However, in the single carcinoma in which a somatic mutation did occur, sparse p53 protein expression was observed with antibody MAb DO-7. In the two tumours with silent mutations at codon 213, one showed similar sparse accumulation of p53 protein product while the other did not. Therefore, in this series, no clear-cut correlation could be demonstrated between immunocytochemical detection of p53 and the occurrence of p53 mutations.

In other neoplasms, mutation in one p53 allele is frequently accompanied by loss of the corresponding normal allele (Baker *et al.*, 1990; Iggo *et al.*, 1990; Prosser *et al.*, 1990; Eccles *et al.*, 1992). By contrast, the cervical carcinomas in this series showed a relatively low frequency of LOH on chromosome 17p (15%), within the range expected as 'background' for any gene locus examined, and in keeping with the relative absence of p53 mutations. Moreover, in the three cases showing allele loss, no mutations were detected in the remaining allele in the four 'hotspot' regions suggesting these may have occurred as a consequence of the general genetic instability exhibited by many tumours. These findings are in keeping with those of Helland *et al.* (1993), who reported allelic imbalance in 11 out of 52 informative cases (22%) at the p53 locus.

Other recent studies have suggested that inactivation of p53 function is fundamental to cervical carcinogenesis (Crook *et al.*, 1991*a*, 1992). It has been proposed that inactivation of the gene product may occur either by p53 gene mutation in HPV-negative tumours, or by complexing with the HPV E6 transforming gene product in HPV-positive tumours. In our studies, only 1 of 11 HPV-negative carcinomas showed evidence of a somatic mutation in any of the four mutational 'hotspots' of the p53 gene. However, we screened only for HPV types 16, 18 and 33, most commonly associated with cervical cancer, and we cannot exclude the possibility that some negative cases may have been positive for other less common high-risk virus types. Our findings are consistent with those of Helland *et al.* (1993), who found no mutations in the p53 gene in all four of their HPV-negative patients.

If increased p53 expression in benign, premalignant and malignant lesions reflects DNA repair, it is surprising that it was so rarely observed. Inactivation of p53 may occur by interaction with the HPV E6 gene product, and it is possible that elevated expression of p53 detected immunocytochemically in HPV-positive tumours could reflect lack of E6 gene expression in these tumours. Other possible mechanisms of p53 inactivation such as alteration in *mdm*-2 gene expression have not been addressed in this study. The product of the *mdm*-2 oncogene binds wild-type p53 *in vitro* (Oliner *et al.*, 1992), and it is possible that its increased expression may account for p53 accumulation in our immunocytochemically positive cases.

Overall, our findings suggest that cervical carcinoma differs from other common neoplasms in the manner in which p53 gene alterations are involved in the carcinogenic process. Mutations in the highly conserved regions of the gene are relatively infrequent and no correlation has been found between p53 mutational status and either LOH on chromosome 17p or the presence or integration of HPV 16, 18 or 33. Finally, the immunohistochemical demonstration of p53 is not a striking feature in the preinvasive or invasive phases of the malignancy.

The authors are grateful to the patients who agreed to participate in this study. We thank clinical and pathology colleagues in Lothian Health Board for assistance with the collection of specimens. We are indebted to Mr J. Lauder and Mr D. Nicholson for technical assistance; to Ms M. Wallace and Ms A. Gallacher of the MRC Human Genetics Unit, Edinburgh, for providing p53 PCR primers; and to Professor David Lane for generously supplying MAb DO-7. We are also grateful to Professor A.H. Wyllie for helpful advice. This research was funded by grants from the Medical Research Council and the Cancer Research Campaign.

### References

ARENDS, M.J., DONALDSON, Y.K., DUVALL, E., WYLLIE, A.H. & BIRD, C.C. (1991). HPV in full thickness cervical biopsies: high prevalence in CIN 2 and CIN 3 detected by a sensitive PCR method. *J. Pathol.*, **165**, 301-309.

BAKER, S.J., PREISINGER, A.C., JESSUP, J.M., PARASKEVA, C., MARKOWITZ, S., WILLSON, J.K.V., HAMILTON, S. & VOGELSTEIN, B. (1990). p53 gene mutations occur in combination with 17p allelic deletions as late events in colorectal tumorigenesis. *Cancer Res.*, **50**, 7717-7722.

BANKS, L., MATLASHEWSKI, G. & CRAWFORD, L. (1986). Isolation of human-p53-specific monoclonal antibodies and their use in the studies of human p53 expression. *Eur. J. Biochem.*, **159**, 529-534.

BANKS, L., EDMONDS, C. & VOUSDEN, K.H. (1990). Ability of HPV 16 E7 protein to bind RB and induce DNA synthesis is not sufficient for efficient transforming activity in NIH3T3 cells. *Oncogene*, **5**, 1383-1389.

BARTEK, J., BARTKOVA, J., VOJTESEK, B., STASKOVA, Z., LUKAS, J., REJTHAR, A., KOVARIK, J., MIDGLEY, C.A., GANNON, J.V. & LANE, D.P. (1991). Aberrant expression of the p53 oncoprotein is a common feature of a wide spectrum of human malignancies. *Oncogene*, **6**, 1699-1703.

BORRESEN, A., HOVIG, E., SMITH-SORENSEN, B., MALKIN, D., LYSTAD, S., ANDERSEN, T.I., NESLAND, J.M., ISSELBACHER, K.J. & FRIEND, S.H. (1991). Constant denaturant gel electrophoresis as a rapid screening technique for p53 mutations. *Proc. Natl Acad. Sci. USA.*, **88**, 8405-8409.

BOSCH, F.X. & MUNOZ, N. (1989). Human papillomavirus and cervical neoplasia: a critical review of the available epidemiological evidence. In *Human Papillomavirus in Cervical Cancer*, IARC Scientific Publication No. 94, Munoz, N., Bosch, F.X. & Jensen, O.M. (eds), pp. 135-151. IARC: Lyon.

BUSBY-EARLE, R.M.C., STEEL, C.M. & BIRD, C.C. (1993). Cervical carcinoma: low frequency of allele loss at loci implicated in other common malignancies. *Br. J. Cancer*, **67**, 71-75.

CARON DE FROMENTEL, C. & SOUSSI, T. (1992). TP53 tumour suppressor gene: a model for investigating human mutagenesis. *Genes Chrom. Cancer*, **4**, 1-15.

CATTORETTI, G., RILKE, F., ANDREOLA, S., D'AMATO, L. & DELIA, D. (1988). p53 expression in breast cancer. *Int. J. Cancer*, **41**, 178-183.

CHIBA, I., TAKASHI, T., NAU, M.M., D'AMICO, D., CURIEL, D.T., MITSUDOMI, T., BUCHHAGEN, D.L., CARBONE, D., PIANTADOSI, S., KOGA, H., REISSMAN, P.T., SLAMON, D.J., HOLMES, E.C. & MINNA, J.D. (1990). Mutations in the p53 gene are frequent in primary, resected non-small cell lung cancer. *Oncogene*, **5**, 1603-1610.

CROOK, T., FISHER, C. & VOUSDEN, K.H. (1991*a*). Modulation of immortalising properties of human papillomavirus type 16 E7 by p53 expression. *J. Virol.*, **65**, 505-510.

CROOK, T., WREDE, D. & VOUSDEN, K.H. (1991*b*). p53 point mutation in HPV negative human cervical carcinoma cell lines. *Oncogene*, **6**, 873-875.

CROOK, T., WREDE, D., TIDY, J.A., MASON, W.P., EVANS, D.J. & VOUSDEN, K.H. (1992). Clonal p53 mutation in primary cervical cancer: association with human-papillomavirus-negative tumours. *Lancet*, **339**, 1070-1073.

DAVIDOFF, A.M., HERNDON, J.E., GLOVER, N.S., KERNS, B.M., PENCE, J.C., IGLEHART, J.D. & MARKS, J.R. (1991). Relation between p53 overexpression and established prognostic factors in breast cancer. *Surgery*, **110**, 259-264.

DEVILEE, P., VAN DEN BROEK, M., KUIPERS-DIJKSHOORN, N., KOLLURI, R., KHAN, P.M., PEARSON, P.L. & CORNELISSE, C.J. (1989). At least four different chromosomal regions are involved in loss of heterozygosity in human breast carcinoma. *Genomics*, **5**, 554-560.

ECCLES, D.M., CRANSTON, G., STEEL, C.M., NAKAMURA, Y. & LEONARD, R.C.F. (1990). Allele losses on chromosome 17 in human epithelial ovarian carcinoma. *Oncogene*, **5**, 1599-1601.

ECCLES, D.M., BRETT, L., LESSELLS, A., GRUBER, L., LANE, D., STEEL, C.M. & LEONARD, R.C.F. (1992). Overexpression of the p53 protein and allele loss at 17p13 in ovarian carcinoma. *Br. J. Cancer*, **65**, 40-44.

FUJITA, M., INOUE, M., TANIZAWA, O., IWAMOTO, S. & ENAMOTO, T. (1992). Alterations of the p53 gene in human primary cervical carcinoma with and without human papillomavirus infection. *Cancer Res.*, **52**, 5323-5328.

FULTS, D., TIPPETS, R.H., THOMAS, G.A., NAKAMURA, Y. & WHITE, R. (1989). Loss of heterozygosity for loci on chromosome 17p in human malignant astrocytoma. *Cancer Res.*, **49**, 6572-6577.

HELLAND, A., HOLM, R., KRISTENSEN, G., KAERN, J., KARLSEN, F., TROPE, C., NESLAND, J.M., BORRESEN, A. (1993). Genetic alterations of the TP53 gene, p53 protein expression and HPV infection in primary cervical carcinomas. *J. Pathol.*, **171**, 105–114.

IGGO, R., GATTER, K., BARTEK, J., LANE, D. & HARRIS, A. (1990). Increased expression of mutant forms of p53 oncogene in primary lung cancer. *Lancet*, **335**, 675–679.

KAELBLING, M., BURK, R.D., ATKIN, N.B., JOHNSON, A.B. & KLINGER, H.P. (1992). Loss of heterozygosity on chromosome 17p and mutant p53 in HPV-negative cervical carcinomas. *Lancet*, **340**, 140–142.

KOHLER, M.F., BERCHUCK, A., DAVIDOFF, A.M., HUMPHREY, P.A., DODGE, R.K., IGLEHART, J.D., SOPER, J.T., CLARKE-PEARSON, D.L., BAST, R.C. & MARKS, J.R. (1992). Overexpression and mutation of p53 in endometrial carcinoma. *Cancer Res.*, **52**, 1622–1627.

LANE, D.P. (1992). p53, guardian of the genome. *Nature*, **358**, 15–16.

LANE, D.P. & BENCHIMOL, S. (1990). p53: oncogene or anti-oncogene? *Genes Dev.*, **4**, 1–8.

MACKAY, J., STEEL, C.M., ELDER, P.A., FORREST, A.P.M. & EVANS, H.J. (1988). Allele loss on short arm of chromosome 17 in breast cancers. *Lancet*, **ii**, 1384–1385.

MIDGLEY, C.A., FISHER, C.J., BARTEK, J., VOJTESEK, B., LANE, D. & BARNES, D.M. (1992). Analysis of p53 expression in human tumours: an antibody raised against human p53 expressed in *Escherichia coli*. *J. Cell Sci.*, **101**, 183–189.

MORI, N., YOKOTA, J., OSHIMURA, M., CAVENEE, W.K., MIZOGUCHI, H., NOGUCHI, M., SHIMOSATO, Y., SUGIMURA, T. & TERADA, M. (1989). Concordant deletions of chromosome 3p and loss of heterozygosity for chromosomes 13 and 17 in small cell lung carcinoma. *Cancer Res.*, **49**, 5130–5135.

NIGRO, J.M., BAKER, S.J., PREISINGER, A.C., JESSUP, J.M., HOSTETTER, R., CLEARY, K., BIGNER, S., DAVIDSON, N., BAYLIN, S., DEVILEE, P., GLOVER, T., COLLINS, F.S., WESTON, A., MODALI, R., HARRIS, C.C. & VOGELSTEIN, B. (1989). Mutations in the p53 gene occur in diverse human tumour types. *Nature*, **342**, 705–708.

OLINER, J.D., KINZLER, K.W., MELTZER, P.S., GEORGE, D.L. & VOGELSTEIN, B. (1992). Amplification of a gene encoding a p53-associated protein in human sarcomas. *Nature*, **358**, 80–83.

O'ROURKE, R.W., MILLER, C.W., KATO, G.J., SIMON, K.J., CHEN, D., DANG, C.V. & KOEFFLER, H.P. (1990). A potential transcriptional activation element in the p53 protein. *Oncogene*, **5**, 1829–1832.

POLLARD, K., LUNNY, D., HOLGATE, C.S., JACKSON, P. & BIRD, C.C. (1987). Fixation, processing and immunochemical reagent effects on preservation of T-lymphocyte surface membrane antigens in paraffin embedded tissue. *J. Histochem. Cytochem.*, **35**, 1329–1338.

PROSSER, J., THOMPSON, A.M., CRANSTON, G. & EVANS, H.J. (1990). Evidence that p53 behaves as a tumour suppressor gene in sporadic breast tumours. *Oncogene*, **5**, 1573–1579.

PURDIE, C.A., O'GRADY, J., PIRIS, J., WYLLIE, A.H. & BIRD, C.C. (1991). p53 expression in colorectal tumors. *Am. J. Pathol.*, **138**, 807–813.

RUSSELL, S.E.H., HICKEY, G.I., LOWRY, W.S., WHITE, P. & ATKINSON, R.J. (1990). Allele loss from chromosome 17 in ovarian cancer. *Oncogene*, **5**, 1581–1583.

SCHEFFNER, M., MUNGER, K., BYRNE, J.C. & HOWLEY, P.M. (1991). The state of the p53 and retinoblastoma genes in human cervical carcinoma cell lines. *Proc. Natl Acad. Sci. USA*, **88**, 5523–5527.

SHEFFIELD, V.C., COX, D.R., LERMAN, L.S. & MYERS, R.M. (1989). Attachment of a 40-base-pair G + C rich sequence (GC-clamp) to genomic DNA fragments by the polymerase chain reaction results in improved detection of single-base changes. *Proc. Natl Acad. Sci. USA*, **86**, 232–236.

TOGUCHIDA, J., ISHIZAKI, K., NAKAMURA, Y., SASAKI, M.S., IKENAGA, M., KATO, M., SUGIMOTO, M., KOTOURA, Y. & YAMAMURO, T. (1989). Assignment of common allele loss in osteosarcoma to the subregion 17p13. *Cancer Res.*, **49**, 6247–6251.

TSAI, Y.C., NICHOLS, P.W., HITI, A.L., WILLIAMS, Z., SKINNER, D.G. & JONES, P.A. (1990). Allelic losses of chromsomes 9, 11 and 17 in human bladder cancer. *Cancer Res.*, **50**, 44–47.

VOGELSTEIN, B., FEARON, E.R., HAMILTON, S.R., KERN, S.E., PREISINGER, A.C., LEPPERT, M., NAKAMURA, Y., WHITE, R., SMITS, A.M.M. & BOS, J.L. (1988). Genetic alterations during colorectal-tumor development. *N. Engl. J. Med.*, **319**, 525–532.

VOJTESEK, B., BARTEK, J., MIDGLEY, C.A. & LANE, D.P. (1992). An immunochemical analysis of human p53: new monoclonal antibodies and epitope mapping using recombinant DNA. *J. Immunol. Methods*, **151**, 237–244.

WYNFORD-THOMAS, D. (1992). p53 in tumour pathology: can we trust immunocytochemistry? (editorial). *J. Pathol.*, **166**, 329–330.

ZUR HAUSEN, H. (1989). Papillomavirus in anogenital cancer: the dilemma of epidemiological approaches (editorial). *J. Natl Cancer Inst.*, **81**, 1680–1682.